# Using Structural Equation Models to Interpret Genome-Wide Association Studies for Morphological and Productive Traits in Soybean [*Glycine max* (L.) Merr.]

**DOI:** 10.3390/plants14193015

**Published:** 2025-09-29

**Authors:** Matheus Massariol Suela, Camila Ferreira Azevedo, Ana Carolina Campana Nascimento, Gota Morota, Felipe Lopes da Silva, Gaspar Malone, Nizio Fernando Giasson, Moysés Nascimento

**Affiliations:** 1 Blueberry Breeding and Genomics Lab, Horticultural Sciences Department, University of Florida, Gainesville, FL 32611, USA; 2 Department of Statistics, Federal University of Viçosa, Viçosa 36570-900, MG, Brazil; 3 Laboratory of Biometry and Bioinformatics, Department of Agricultural and Environmental Biology, Graduate School of Agricultural and Life Sciences, The University of Tokyo, Bunkyo, Tokyo 113-8657, Japan; 4 Department of Agronomy, Federal University of Viçosa, Viçosa 36570-900, MG, Brazil; 5 GDM Seeds, Cambé 86183-751, PR, Brazil

**Keywords:** genome-wide association study, structural equation model, Bayesian network, single nucleotide polymorphism, *Glycine max* (L.) Merr

## Abstract

Understanding trait relationships is fundamental in soybean breeding because the goal is to maximize simultaneous gains. Standard multi-trait genome-wide association studies (MT-GWAS) identify variants linked to multiple traits but fail to capture phenotypic structures or interrelations. Structural Equation Models (SEM) account for covariances and recursion, enabling the decomposition of single nucleotide polymorphism (SNP) effects into direct or indirect components and identifying pleiotropic regions. We applied SEM to analyze morphology (pod thickness, PT) and yield traits (number of pods, NP; number of grains, NG; hundred-grain weight, HGW). The dataset comprised 96 soybean individuals genotyped with 4070 SNP markers. The phenotypic network was constructed using the hill-climbing algorithm, a class of score-based methods commonly applied to learn the structure of Bayesian networks, and structural coefficients were estimated with SEM. According to coefficient signs, we identified negative interrelationships between NG and HGW, and positive ones between NP and NG, and HGW and PT. NG, HGW, and PT showed indirect SNP effects. We also found loci jointly controlling traits. In total, 46 candidate genes were identified: 7 associated exclusively with NP and 4 associated with NG. An additional 15 genes were common to NP and NG, 3 were common to NP and HGW, 6 were common to NG and HGW, and 11 were common to NP, NG, and HGW. In summary, SEM-GWAS revealed novel relationships among soybean traits, including PT, supporting breeding programs.

## 1. Introduction

Soybean is one of the five crops that dominate global agriculture, along with maize, wheat, cotton, and rice [1]. It is one of the most important commodities in global trade [2]. Brazil is the largest producer of soybeans in the world, producing 129.5 million metric tons, or approximately 36% of global production [1]. Soybean meal is closely linked to the food supply through direct and indirect consumption as an excellent feed supplement, especially for monogastric animals [3]. Soybean oil is highly versatile, with applications in the food and beverage, wax, construction, cosmetics, plastics, and fuel industries [4].

Several studies using the univariate genome-wide association study (GWAS) approach have been used to identify genomic regions associated with important soybean traits, such as disease resistance [5,6,7,8], seed protein and oil content [9,10,11,12,13], salt tolerance [14,15], physiological-related traits [16,17,18], and agronomic traits [10,19,20,21,22,23,24,25]. Although the univariate GWAS methodology is the most commonly used in a breeding program, multiple traits are often studied simultaneously. Another approach that allows the detection of genomic regions involved in linkage or pleiotropy is multivariate GWAS (MTM-GWAS). However, both univariate and multivariate GWAS approaches do not allow us to study the interrelationships among the traits.

Given these limitations, some multivariate GWAS approaches have been developed to assess the associations among the traits. Shim et al. [26] proposed a methodology based on the Bayes factor (mvBIMBAM). Gianola and Sorensen [27], Momen et al. [28], and Wang et al. [18] used structural equation modeling in the context of GWAS (SEM-GWAS). SEM makes it possible to interpret results differently from a multiple trait model (MT), which only captures covariances between variables without considering the existence of recursion. In contrast to this phenomenon, SEM explores the interrelationships between variables, in which one trait can be considered a predictor of another trait [27]. Thus, by uniting SEM to GWAS (SEM-GWAS), it becomes possible to model the associations between traits and quantitative trait loci (QTL) by decomposing single nucleotide effects of polymorphisms (SNPs) in a trait into direct or indirect components and also to identify genomic regions with pleiotropic effects [29,30,31].

For this type of methodology, it is common to use Bayesian networks (BNs) to generate connections between variables based on the theory of Direct Acyclic Graph (DAG) and conditional independence; that is, it is used as a hypothesis-generating tool in determining the causal nature of the connections found, as the interrelations between the traits are not always known *a priori* [32]. Several works have already been proposed based on this type of logic [29,30,31,33,34,35]. From the best network hypothesis created, the SEM methodology was used to identify the structural coefficient.

The SEM-GWAS approach can account for trait interactions and partition single nucleotide polymorphism (SNP) effects from the trait itself (direct) and from other related traits (indirect). Thus, the objectives of this study were to (1) estimate genetic components for important traits in soybeans, (2) use Bayesian network approaches to estimate a phenotypic network reflecting the interrelationships among traits, and (3) use SEM-GWAS approaches to estimate direct and indirect SNP effects.

## 2. Results

### 2.1. Descriptive Statistics

Descriptive statistics for the number of pods per plant (NP), number of grains per plant (NG), hundred-grain weight (HGW), and pod thickness (PT) were calculated across all genotypes (Table 1). On average, the plants produced 53.47 (±11.72) $pods.{plant}^{-1}$, 111.40 (±22.54) $grains.{plant}^{-1}$, and 14.57 (±3.04) $g.{100\;grains}^{-1}$, and the thickness of the pod was 6.41 (±0.87) mm. These values summarize the phenotypic variability present in the population and provide the basis for subsequent genetic analyses.

### 2.2. Genetic Parameters

To estimate genetic parameters, we fitted a Bayesian multivariate mixed model including additive genomic relationships among genotypes and independent residuals for the four traits: number of pods per plant (NP), number of grains per pod (NG), hundred-grain weight (HGW), and total pod weight (PT). Narrow-sense heritability was calculated from the ratio of additive genetic to total variance, and genomic and residual correlations were derived from the posterior distributions of the additive and residual covariance matrices. Significance was assessed from the 95% highest posterior density (HPD) intervals, where estimates excluding zero were considered significant.

According to Table 2, narrow-sense heritability was moderate to high for all traits, with posterior means (and 95% HPD) of 0.89 (0.73–1.00) for NP, 0.79 (0.44–1.00) for NG, 0.39 (0.14–0.67) for HGW, and 0.45 (0.24–0.66) for PT. Genomic correlations were consistently significant, ranging from strong positive values, such as 0.96 (0.82–1.00) between NP and NG, to strong negative values, such as –0.88 (–0.99 to –0.68) between NG and PT. In contrast, only the residual correlation between HGW and PT, 0.59 (0.35–0.80), was significant, indicating that most trait associations arise from shared genetic rather than environmental effects.

### 2.3. Bayesian Network Structure

To identify conditional dependencies among traits, we inferred a Bayesian network using a score-based hill-climbing algorithm. The algorithm searched the space of possible directed acyclic graphs (DAGs) and retained the structure with the highest posterior score. Edge strength and direction were estimated from 50,000 bootstrap samples of the posterior distribution.

**Figure 1:**
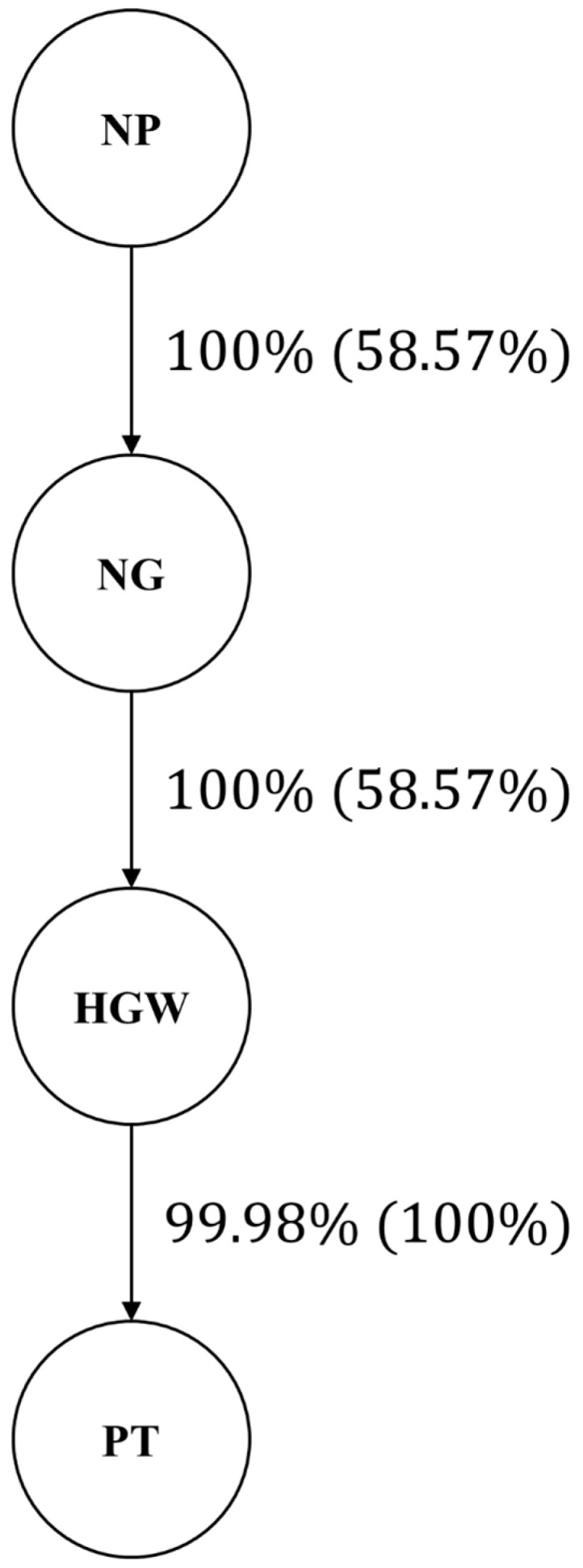
Path network via the HC algorithm from 50,000 bootstrap samples. Values outside the parentheses represent strength (the percentage of bootstrap samples that had an arc), and values inside the parentheses represent direction (the percentage of bootstrap samples in which a given direction of arcs occurred). NP: number of pods; NG: number of grains; HGW: hundred-grain weight; PT: pod thickness.

The resulting network (Figure 1) showed a directed edge from number of pods per plant (NP) to number of grains per pod (NG), with 100% edge strength and 58.57% directional support. A second edge linked NG to hundred-grain weight (HGW) with the same strength and directional support, and a third edge connected HGW to total pod weight (PT), with 99.98% strength and full (100%) directional support. This topology indicates that NP acts as the primary upstream trait, NG is directly influenced by NP, HGW is directly influenced by NG and indirectly by NP through NG, and PT is directly influenced by HGW while also receiving indirect effects from NP and NG.

Model fit was evaluated by the change in the Bayesian information criterion (BIC) when individual edges were removed from the best-scoring network (Table 3). A larger increase in the BIC after removing an edge indicates a greater contribution of that path to the overall model.

The largest effect was observed for the edge from number of pods per plant (NP) to number of grains per pod (NG): deleting this connection increased the BIC by 35.8808, highlighting it as the most influential link in the phenotypic network.

Removing the edge from NG to hundred-grain weight (HGW) and the edge from HGW to total pod weight (PT) produced smaller, though still meaningful, BIC increases of 13.1780 and 25.9670, respectively, indicating moderate contributions of these paths to the network structure.

### 2.4. Structural Equation Model

Unlike the univariate GWAS and MTM-GWAS models, the graphical structure inferred from Bayesian networks and SEM explains how phenotypes can be related to each other, directly or indirectly. It allows researchers to understand how changes in one trait might directly influence another, or how they might be connected through a chain of intermediate effects. In addition, this approach can reveal potential causal pathways or correlated factors that connect the phenotypes. This can provide clues about the underlying biological mechanisms that explain how the traits are related.

**Figure 2:**
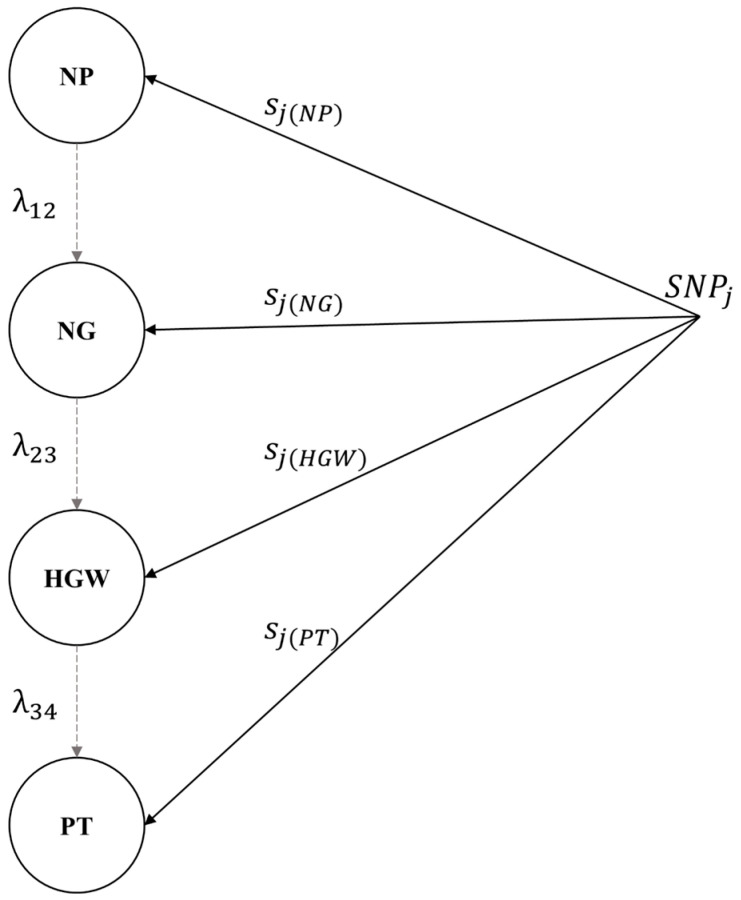
Path network of the SNP markers’ effects. NP: number of pods; NG: number of grains; HGW: hundred-grain weight; PT: pod thickness. The gray dashed arcs indicate the direction of the interrelations.$\lambda_{12}$ : NP → NG; $\lambda_{23}$: NG → HGW; $\lambda_{34}$: HGW → PT. The black arcs indicate the direct effect of the *j*th SNP.

The estimated values of the structural coefficients represent the average increase in the downstream phenotype for a one-unit increase in the upstream phenotype (Table 4). We observed that the paths between NP and NG and between HGW and PT were positive (0.00006 and 0.00697, respectively), whereas the path between NG and HGW was negative (−0.05450). The magnitude of the estimated structural coefficient values was small, suggesting that the role of upstream traits in mediating SNP effects of downstream traits was marginal. Increasing NP, NG, and HGW by 1 unit each resulted in an average increase of 0.00006, 0.00697, and −0.05450 units in NG, HGW, and PT, respectively. Figure 2 shows the relationship between the inferred phenotypic network and the SNPs, where $s_{j(NP)}$, $s_{j(NG)}$, $s_{j\left(HGW\right),}$ and $s_{j(PT)}$ represent the direct effects of the SNPs on NP, NG, HGW, and PT, respectively, and $\lambda_{12}$, $\lambda_{23},$ and $\lambda_{34}$ represent the structural coefficients associated with the phenotypic network.

### 2.5. Partitioning of SNP Effects

**Figure 3:**
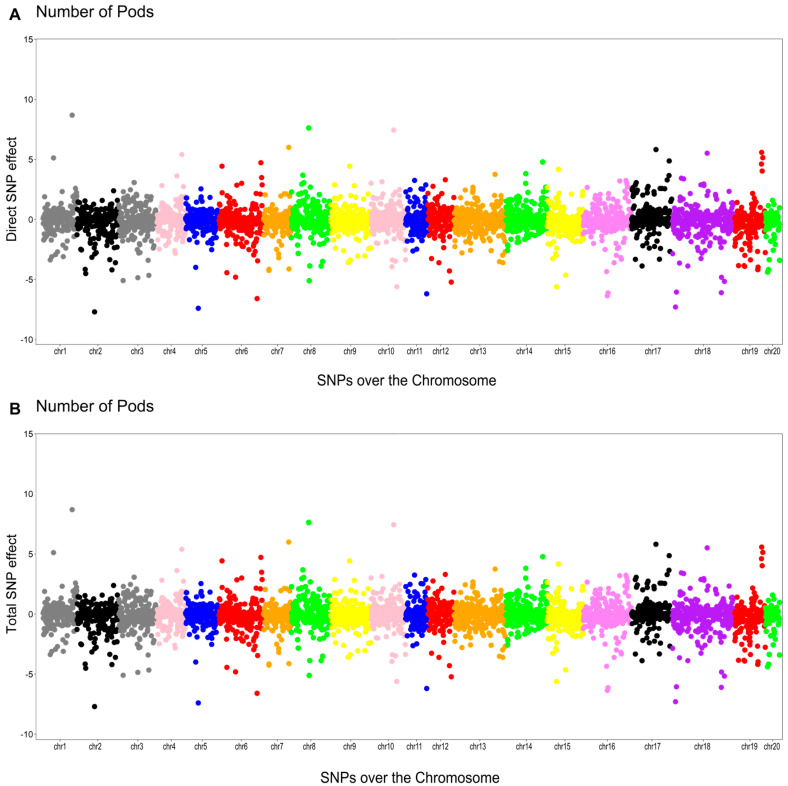
Manhattan plots for the direct (**A**) and total (**B**) SNP effects associated with the number of pods (NP) via the SEM-GWAS approach.

**Figure 4:**
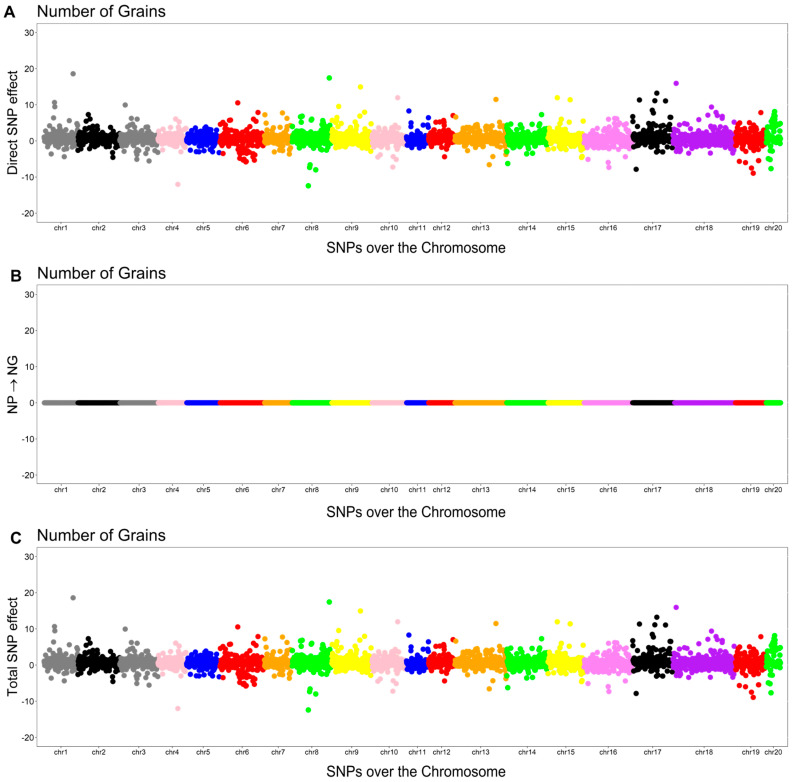
Manhattan plots for direct (**A**), indirect (**B**), and total (**C**) SNP effects associated with the number of grains (NG) via the SEM-GWAS approach. NP refers to the number of pods.

**Figure 5:**
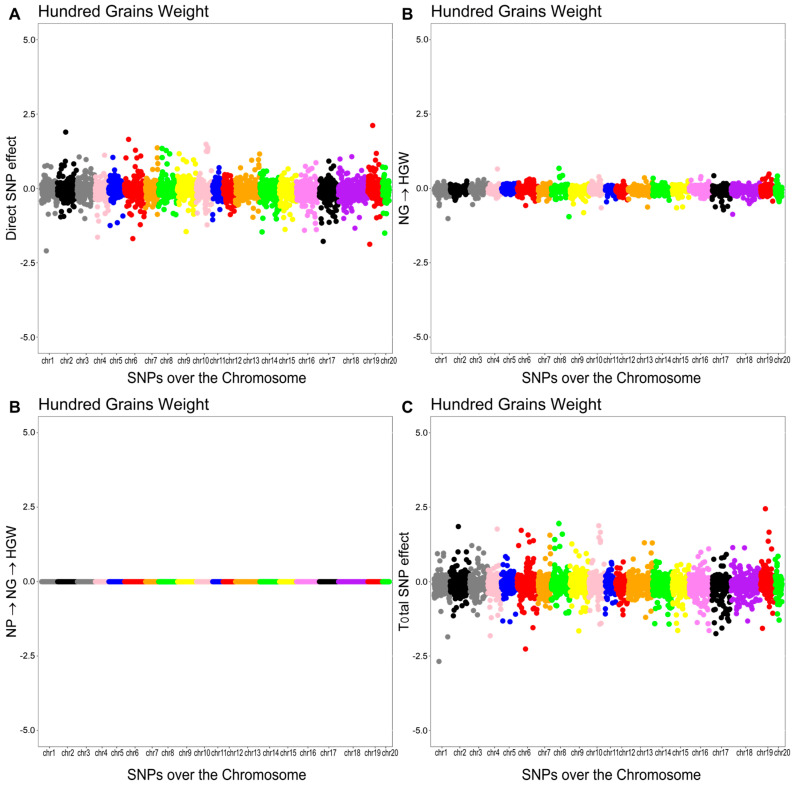
Manhattan plots for direct (**A**), indirect (**B**), and total (**C**) SNP effects associated with hundred-grain weight (HGW) via the SEM-GWAS approach. NP and NG refer to number of pods and number of grains, respectively.

**Figure 6:**
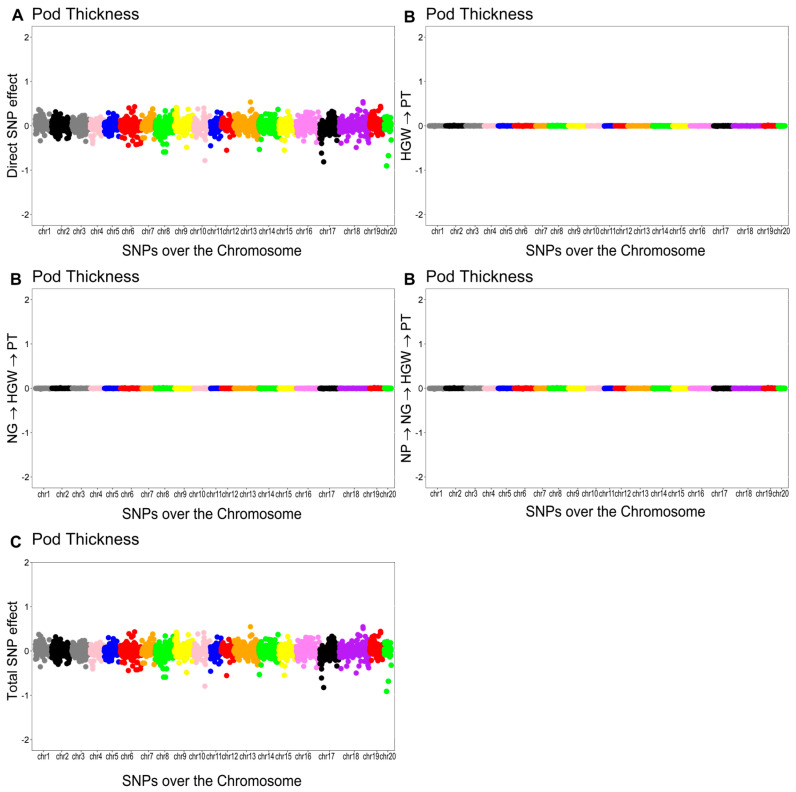
Manhattan plots for direct (**A**), indirect (**B**), and total (**C**) SNP effects associated with pod thickness (PT) via the SEM-GWAS approach. NP, NG, and HGW refer to number of pods, number of grains, and hundred-grain weight, respectively.

As shown in Figure 3, Figure 4, Figure 5 and Figure 6, the effects of SNPs could be decomposed into direct, indirect, and total effects for each characteristic using the SEM-GWAS approach.

#### 2.5.1. Number of Pods (NP)

The phenotypic network did not identify any mediator trait for NP (Figure 2). In this case, the total effect of the jth SNP on NP consists of its direct effect:

$${Direct}_{s_{j}\to {y1}_{NP}}=s_{j({y1}_{NP})}$$

$${Total}_{s_{j}\to {y1}_{NP}}={Direct}_{s_{j({y1}_{NP})}}=s_{j({y1}_{NP})}$$

The Manhattan plots of the direct (A) and total (B) SNP effects on NP are presented in Figure 3.

#### 2.5.2. Number of Grains (NG)

In addition to the direct effect of the jth SNP associated with NG, there was an indirect effect mediated by NP ($\lambda_{12}=0.00006$). Thus, the total effect was calculated by summing the direct and indirect effects.

$${Direct}_{s_{j}\to {y2}_{NG}}=s_{j({y2}_{NG})}$$

$${Indirect(1)}_{s_{j}\to {y2}_{NG}}=\lambda_{12}s_{j({y1}_{NP})}$$

$${Total}_{s_{j}\to {y2}_{NG}}={Direct}_{s_{j}\to {y2}_{NG}}+{Indirect(1)}_{s_{j}\to {y1}_{NP}}=s_{j({y2}_{NG})}+\lambda_{12}s_{j({y1}_{NP})}$$

The Manhattan plots of the direct (A), indirect (B), and total (C) SNP effects on NG are presented in Figure 4.

#### 2.5.3. Hundred-Grain Weight (HGW)

The overall effect of the SNP on HGW was decomposed into a direct effect and two indirect effects mediated by NG and NP. The structural coefficients for the indirect effects were $\lambda_{23}$ (0.0018) for NG and $\lambda_{12}\times \lambda_{23}$ (0.00006×−0.05450=−0.00000327) for NP. The total effect of the jth SNP on HGW was equal to the sum of the direct and indirect effects.

$${Direct}_{s_{j}\to {y3}_{HGW}}=s_{j({y3}_{HGW})}$$

$${Indirect(1)}_{s_{j}\to {y3}_{HGW}}=\lambda_{23}s_{j({y2}_{NG})}$$

$${Indirect(2)}_{s_{j}\to {y3}_{HGW}}=\lambda_{12}{\lambda_{23}s}_{j({y1}_{NP})}$$

$${Total}_{s_{j}\to {y3}_{HGW}}={Direct}_{s_{j}\to {y3}_{HGW}}+{Indirect(1)}_{s_{j}\to {y3}_{HGW}}+{Indirect(2)}_{s_{j}\to {y3}_{HGW}}\;=s_{j({y3}_{HGW})}+\lambda_{23}s_{j({y2}_{NG})}+\lambda_{12}{\lambda_{23}s}_{j({y1}_{NP})}$$

The Manhattan plots of the direct (A), indirect (B), and total (C) SNP effects on HGW are presented in Figure 5.

#### 2.5.4. Pod Thickness (PT)

The overall effect of the SNP on PT was decomposed into a direct effect and three indirect effects mediated by HGW, NG, and NP. The structural coefficients for the indirect effects were $\lambda_{34}$ (0.0018) for HGW, $\lambda_{23}\times \lambda_{34}$ (−0.05450×0.00697=−0.0003799) for NG, and ${\lambda_{12}\times \lambda_{23}\times \lambda }_{34}$ (0.00006×−0.05450×0.00697=−0.000000022) for NP. The total effect of the jth SNP on PT was equal to the sum of the direct and indirect effects.

$${Direct}_{s_{j}\to {y4}_{PT}}=s_{j({y4}_{PT})}$$

$${Indirect(1)}_{s_{j}\to {y4}_{PT}}=\lambda_{34}s_{j({y3}_{HGW})}$$

$${Indirect(2)}_{s_{j}\to {y4}_{PT}}=\lambda_{23}{\lambda_{34}s}_{j({y2}_{NG})}$$

$${Indirect(3)}_{s_{j}\to {y4}_{PT}}={\lambda_{12}\lambda }_{23}{\lambda_{34}s}_{j({y1}_{NP})}$$

$${Total}_{s_{j}\to {y4}_{PT}}={Direct}_{s_{j}\to {y4}_{PT}}+{Indirect(1)}_{s_{j}\to {y4}_{PT}}+{Indirect(2)}_{s_{j}\to {y4}_{PT}}+{Indirect(3)}_{s_{j}\to {y4}_{PT}}\;=s_{j(PT)}+\lambda_{34}s_{j({y3}_{HGW})}+\lambda_{23}{\lambda_{34}s}_{j({y2}_{NG})}+{\lambda_{12}\lambda }_{23}{\lambda_{34}s}_{j({y1}_{NP})}$$

The Manhattan plots of the direct (A), indirect (B), and total (C) SNP effects on PT are presented in Figure 6.

### 2.6. Integration of Structural Equation Modeling and Genome-Wide Association Study (SEM-GWAS)

In total, 46 SNPs were statistically significant (q-value < 0.01) for the traits studied (Table S5). Of those, 36, 36, and 20 SNPs were associated with NP, NG, and HGW, respectively. No significant SNP was found for PT. All information on significant SNPs, such as the chromosome to which it belongs, position, q-value, gene, auto defline, gene atlas description, GO, traits, and some previously reported references, is illustrated in Table S5.

For NP, relevant GO terms identified include inactive shikimate kinase, serine-threonine protein kinase, GDT1-like protein, polyglutamine-binding, protein DA1-related 2, β-1, 3-glucanase-like protein, DNAJ homolog, fructose-bisphosphate aldolase, amino acid transporter, protein tyrosine kinase, squamosa promoter-binding, xenobiotic monooxygenase, reverse transcriptase, RNA-binding proprotein convertase subtilisin, NADPH oxidase, DNA mismatch repair, Acyl-CoA n-acyltransferase, dynamin, small heat-shock, ribosomal biogenesis, acylglycerol lipase, snare, phosphatase, U3 small nucleolar, AP-4 complex, ABC transporter, WW domain, proprotein convertase, and HNH endonuclease. For NG, relevant GO terms identified include inactive shikimate kinase, MYB-like DNA-binding, glycine-rich protein, ubiquitin carboxyl-terminal hydrolase, ABC transporter, dentin sialophosphoprotein, threonine-protein kinase SRK-related, monothiol glutaredoxin, and mono-ADP-ribosyltransferase. In Figures S1 and S2, it is possible to observe the number of QTLs found for each GO term.

## 3. Discussion

### 3.1. Genetic Parameters

The heritability estimates for NP and NG were similar to those found in the literature. Our heritability estimate for HGW differed from those found in the literature, possibly because we used a different estimation method. No heritability results were found in the literature for PT. According to Aditya et al. [36], using a database of 31 genotypes of *G*. *max* (L.) Merrill, a heritability estimate of 0.81 was found for NP. Ghiday et al. [37], using 22 promising genotypes from IITA/Nigeria, found a heritability estimate of 0.93 for NP. Del Conte et al. [38], using 34 $F_{1}$ soybean populations in a path analysis, found a heritability for NP of 0.83. For NG, Ghiday et al. [37] reported a heritability of 0.98. Similarly, Del Conte et al. [38] and de Albuquerque et al. [39] reported heritability estimates of 0.70 and 0.59, respectively. For HGW, Bisinotto et al. [40] reported a heritability estimate of 0.77 using 31 lines from a breeding program.

The genomic correlation estimates were consistent with those found in the literature. According to Del Conte et al. [38] and Li et al. [41], the genetic correlation estimates between NP and NG were 0.87 and 0.88, respectively. Li et al. [41] reported genetic correlations of −0.25 and −0.29 between NP and HGW and between NG and HGW, respectively. Silva et al. [42] found a correlation of −0.95 between NG and HGW. The signs of the correlations were identical to those found in this work. However, their estimates differed in magnitude, which could be due to the population and environmental differences from the data in this work. No study was found that showed a correlation with PT.

The occurrence of significant genetic correlations, a fact observed among all characteristics, may indicate a hypothesis that the characteristics may be indicators of one of the others. However, genetic manifestation can be recommended for both linkage disequilibrium and pleiotropy. According to our results, we noticed that the most important effects were direct ones; thus, it can be inferred that the correlations may have been the result of existing pleiotropy and, therefore, it may be very difficult to partition the total effect of the QTL into direct and indirect effects [43].

### 3.2. Integration of Structural Equation Modeling and Genome-Wide Association Study (SEM-GWAS)

The structural equation modeling framework applied here extends multivariate mixed models that are inherently suited for multi-trait genomic data. The estimation proceeds trait by trait within the specified network, keeping overall computational requirements manageable. Previous SEM-GWAS applications illustrate this scalability, with Suela et al. [29] analyzing 21,211 SNPs, Pegolo et al. [30] working with 37,519 SNPs, and Momen et al. [31] including roughly 700,000 SNPs, all reporting successful model fitting without the need for specialized high-performance computing. Within this framework, the component that typically demands the longest runtime is the Markov chain Monte Carlo (MCMC) sampling used to estimate posterior distributions. Although the experimental panel in the present study is compact, statistical corrections were applied to control population structure and relatedness, ensuring that the genetic diversity within the genotypes was effectively captured for association analysis, and the specific strategies adopted to address this smaller population are detailed in the Materials and Methods section.

MTM-GWAS is widely applied, but it remains essentially associative and does not allow the separation of direct genetic effects from those mediated by other phenotypes. Consequently, a single signal may reflect true pleiotropy or merely the indirect influence of an intermediate trait, complicating biological interpretation [44]. Furthermore, studies have shown that different multivariate implementations can inflate type I error rates under deviations from normality or case–control imbalance, thereby reducing statistical robustness [45,46].

By explicitly incorporating causal networks and decomposing the total effect of each SNP into direct and indirect components, SEM-GWAS addresses these limitations and delivers mechanistic insights that MTM-GWAS, by design, cannot achieve. The indirect SNP effects uncovered within the trait network are particularly relevant for breeding programs, as they raise the question of how such effects can be translated into practical selection decisions. As emphasized by Suela et al. [35], statistical equivalence between models does not necessarily imply biological equivalence. Breeding values estimated with traditional multivariate models (MTMs) capture all additive genetic effects—both direct and indirect—arising from pleiotropy, linkage disequilibrium, and shared environmental influences. In contrast, structural equation modeling (SEM) distinguishes direct genetic effects from those mediated through other traits, generating breeding values that are effectively corrected for causal relationships.

This distinction is critical when the objective is to identify precise targets of selection within complex trait networks. While MTM breeding values convey the overall genetic merit required for selection, the structural coefficients and indirect effects estimated by SEM reveal intermediate traits that can be strategically manipulated to maximize genetic gain [35]. In recurrent selection schemes or index-based breeding strategies, such information supports the construction of weighted selection indices that prioritize key mediating traits, enabling more efficient phenotypic and genotypic interventions without the need to redesign the analytical pipeline. Distinguishing direct from indirect genetic effects, therefore, not only refines biological interpretation but also provides actionable guidance for accelerating genetic improvement in complex trait networks [35].

Premature pod opening (PT) of immature soybean pods under water deficits causes significant yield losses across Brazil and globally. Palharini [47] reported that this phenomenon is influenced by genotype and water stress, impacting traits such as the number of pods (NP), number of grains (NG), and hundred-grain weight (HGW). To explore these relationships, we constructed a Bayesian network to model the phenotypic interactions among NP, NG, HGW, and PT, revealing both favorable and unfavorable links associated with pod-opening susceptibility. This phenotypic network was incorporated into a structural equation modeling-based, genome-wide association study (SEM-GWAS) to dissect the SNP effects into direct and indirect contributions within the network.

The SEM-GWAS analysis identified positive direct effects from NP to NG and from HGW to PT, as well as indirect effects from NP to HGW and PT, and from NG to HGW and PT. Positive interrelationships indicate that a higher NP increases NG, and a higher HGW enhances PT, while negative interrelationships suggest that a higher NG reduces HGW (Table 4). Notably, HGW exhibited a substantial indirect effect from NG, suggesting that genetic regulation of seed weight is mediated by grain number, likely through nutrient allocation or stress response pathways. Direct SNP effects were generally stronger than indirect effects for NG and PT, but HGW showed significant indirect effects from NG, underscoring the interconnectedness of these traits (Table 4). Several quantitative trait loci (QTLs) regulated multiple traits, indicating multifunctionality (Table S5), consistent with findings in soybeans [12,22] and rice [31] but contrasting with studies reporting no such pleiotropy [29].

To address the genetic basis of these traits, we analyzed the functions of 46 candidate genes associated with NP, NG, and HGW, several of which control multiple traits simultaneously (Table S5), as previously reported [41,48,49,50,51,52,53,54,55,56,57,58,59,60,61]. To provide a deeper functional interpretation, we linked these genes to specific pathways in soybean physiology and abiotic stress tolerance, particularly water deficits, which exacerbate premature pod opening [47]. Below, we discuss key candidate genes and their roles in these pathways.

*Glyma.01G052600*, a serine-threonine protein kinase, participates in the abscisic acid (ABA) signaling pathway and is crucial for drought stress responses and seed size regulation. Liu et al. [62] showed that this kinase, termed Novel Seed Size (NSS), regulates cell expansion under stress, influencing seed size. Its association with NP, NG, and HGW suggests it coordinates pod and seed development under water deficits by upregulating stress-responsive genes, such as RD29A and DREB, through MAPK cascades [63]. This supports the positive NP-NG relationship, where enhanced stress tolerance promotes higher grain production, potentially reducing pod-opening susceptibility.

*Glyma.02G251800*, a protein DA1-related 2 gene, is involved in the ubiquitin–proteasome pathway, regulating cell proliferation and organ size. Zhao et al. [64] linked DA1 to adaptive radiation in soybeans, while Li et al. [65] described it as a ubiquitin receptor influencing a wide range of cellular processes [66], including cell cycle control [67], abnormal protein degradation [68,69], hormonal signaling via auxin and gibberellin [70,71], and resistance to abiotic stresses [70,72,73]. Its association with NP indicates a role in pod formation by modulating cell division under water-limited conditions, contributing to reduced PT.

*Glyma.02G253800*, a β-1,3-glucanase-like protein, is involved in callose metabolism, a critical pathway for cell wall remodeling that influences pod dehiscence. Callose deposition, regulated by β-1,3-glucanases, strengthens pod cell walls under drought stress, reducing opening susceptibility [74,75,76]. Its association with NP suggests it maintains pod integrity, consistent with Palharini’s findings on water deficit-induced pod opening [47].

*Glyma.03G183900*, a MYB-like DNA-binding protein, regulates stress-responsive pathways, including flavonoid biosynthesis and secondary cell wall formation. Du et al. [77] reported that MYB transcription factors modulate legume-specific nodulation and stress responses. Its association with NG and HGW indicates that it influences seed development and weight by regulating cell-wall reinforcement, potentially mitigating pod dehiscence under water stress.

*Glyma.04G086600*, an amino acid transporter, supports nutrient allocation pathways by transporting amino acids for seed filling [78]. The significant indirect effect of NG on HGW (Table 4) likely reflects this gene’s role in resource allocation, where disruptions under water deficits reduce seed weight and increase PT susceptibility.

*Glyma.04G239000*, a leucine-rich repeat (LRR) protein, is part of the receptor-like kinase (RLK) signaling pathway, regulating growth and stress responses [79]. Its association with NP, NG, and HGW suggests a broad role in coordinating pod and seed development under drought through stress signaling cascades that enhance tolerance.

*Glyma.05G019000*, a squamosa promoter-binding-like protein, regulates developmental transitions and stress responses via jasmonic acid and ethylene pathways [80,81,82,83,84,85,86]. Its association with NP indicates that it controls pod formation by modulating reproductive development timing, which is crucial for minimizing PT under water stress.

*Glyma.09G252200*, a monothiol glutaredoxin, maintains redox homeostasis through glutathione metabolism, protecting developing pods and seeds from oxidative stress under water deficits [87]. Its association with NG and HGW highlights its role in supporting seed development, contributing to the indirect effects observed in the phenotypic network.

*Glyma.12G242300*, a dynamin gene, is involved in vesicle trafficking and cell membrane dynamics, and it is essential for cell growth and stress adaptation [88,89]. Its association with NP and NG suggests that it supports pod and grain development under drought stress.

The multifunctionality of QTLs controlling multiple traits (Table S5) likely reflects pleiotropy or tight linkage within pathways such as ABA signaling, ubiquitin-mediated cell cycle control, callose metabolism, and redox homeostasis. These pathways converge to regulate the phenotypic network underlying pod opening and seed traits under water deficits. For instance, the indirect effect of NG on HGW may involve nutrient allocation [78] or redox homeostasis [87], which are disrupted under stress, affecting seed weight and PT.

## 4. Materials and Methods

### 4.1. Phenotypic Data and SNP Genotyping

The phenotypic data of the genotypes from GDM seeds were kindly provided by the soybean breeding program of the Federal University of Viçosa (in Portuguese, the Universidade Federal de Viçosa—UFV). The experiment was conducted in a greenhouse located in the city of Viçosa—MG (Brazil) (lat: 20°45′17″ S; length: 42°52′57″ W) and planted from January to May 2018. The design used was randomized blocks, with 96 soybean genotypes evaluated in 3 blocks. The experimental plot consisted of a plant grown in a pot with a volume of seven liters, containing a substrate prepared with a mixture of soil, sand, and animal manure, in a ratio of 3:1:2. The substrate was corrected based on chemical analysis, using crop extraction values for production of 3000 ${kg.ha}^{-1}$ [90]. The remaining cultural treatments were carried out as recommended for soybean cultivation [90]. The average temperature and humidity of the greenhouse, which were monitored with the aid of a digital thermohygrometer (model K29-5070H-Kasvi), varied from 22.5 to 34 °C and from 35.5 to 40.5%, respectively. The genotypes evaluated came from different soybean genetic improvement companies (widely planted in Brazil) with great genetic variation in their traits, including different transgenic events (conventional, Roundup Ready and Intacta RR2BT), growth types (determinate, semi-determinate, and indeterminate), and maturity groups (five to nine). Although the experimental panel is compact, statistical corrections were applied to control population structure and relatedness, ensuring that the diversity present was effectively captured for the association analysis. The percentage of immature pods opened per plant was evaluated at the R6 development stage (green pods with full grains) based on the total number of pods. Four traits were evaluated in this work: NP—number of pods (${pods.plant}^{-1}$), NG—number of grains (${grains.plant}^{-1}$), HGW—hundred-grain weight (g), and PT—pod thickness (mm). For the association analysis, a panel of SNP markers was used, with 4070 informative markers for the set of genotypes. The SNP quality control parameters used were call rate (≥95%) and minor allele frequency (<5%). The genotyping data of the genotypes were provided by GDM Seeds. The DNA of 81 and 18 young trifoliate soybean genotypes was extracted using the hexadecyltrimethyl ammonium bromide method [91]. The genotyping was made via genotyping by sequencing (GBS) at the Institute for Genome Diversity at Cornell University (Ithaca, NY) and the Biotechnology Center at the University of Wisconsin–Madison (Wisconsin, USA) [92]. The DNA library was prepared using the restriction enzyme ApeKI [93], and the DNA sequencing was performed using 90-bp according to the GBS protocol by an Illumina HiSeq [93,94].

### 4.2. Phenotypic Data Analysis

Prior to GWAS, the phenotypic values were adjusted for systematic effects according to the following statistical model:

(1) y=Xu+Zg+Wr+e,

where y is the vector of observed phenotypes, u and r are the vectors of overall mean (fixed effect) and between block effect (fixed effect), respectively, and g and e are the vectors of genotypic effects (random effect) and model residuals (random effect), respectively. X, Z, and W are the incidence matrices relating u, g, and r, respectively. The vectors g and e assume a normal distribution $g∼N\left(0,{I\sigma }_{g}^{2}\right)$and $e∼N(0,{I\sigma }_{e}^{2})$, where $\sigma_{g}^{2}$ and $\sigma_{e}^{2}$ are the variance components, and I is an identity matrix. The adjusted phenotypic values were obtained as the sum of the estimates of random effects $\hat{g}$ and $\hat{e}$; that is, $y^{*}=Z\hat{g}+\hat{e}$ [95]. These analyses were conducted using the R software environment [96].

### 4.3. Bayesian Multi-Trait Genomic Best Linear Unbiased Prediction Model

The corrected phenotypes were used in the following Bayesian multi-trait genomic best linear unbiased prediction model.

(2) $$\left[\begin{array}{c} y_{NP}^{*}\\ y_{NG}^{*}\\ y_{HGW}^{*}\\ y_{PT}^{*} \end{array}\right]=\left[\begin{array}{cccc} X_{NP} & 0 & 0 & 0\\ 0 & X_{NG} & 0 & 0\\ 0 & 0 & X_{HGW} & 0\\ 0 & 0 & 0 & X_{PT} \end{array}\right]\left[\begin{array}{c} b_{NP}\\ b_{NG}\\ b_{HGW}\\ b_{PT} \end{array}\right]+\left[\begin{array}{cccc} Z_{NP} & 0 & 0 & 0\\ 0 & Z_{NG} & 0 & 0\\ 0 & 0 & Z_{HGW} & 0\\ 0 & 0 & 0 & Z_{PT} \end{array}\right]\left[\begin{array}{c} g_{NP}\\ g_{NG}\\ g_{HGW}\\ g_{PT} \end{array}\right]+\left[\begin{array}{c} e_{NP}\\ e_{NG}\\ e_{HGW}\\ e_{PT} \end{array}\right]$$

where ${\left[\begin{array}{cccc} y_{NP}^{*} & y_{NG}^{*} & y_{HGW}^{*} & y_{PT}^{*} \end{array}\right]}^{t}$ is the vector of adjusted phenotypes for t traits (t=4), $X_{NP}$, $X_{NG}$, $X_{HGW}$, and $X_{PT}$ are the incidence matrices (only including the intercepts) for t traits; ${\left[\begin{array}{cccc} b_{NP} & b_{NG} & b_{HGW} & b_{PT} \end{array}\right]}^{t}$ is the vector of the mean effects for t traits; $Z_{NP}$, $Z_{NG}$, $Z_{HGW}$, and $Z_{PT}$ are the incidence matrices associating $g_{NP}$, $g_{NG}$, $g_{HGW}$, and $g_{PT}$ with $y_{NP}^{*}$, $y_{NG}^{*}$, $y_{HGW}^{*}$, and $y_{PT}^{*}$; ${\left[\begin{array}{cccc} g_{NP} & g_{NG} & g_{HGW} & g_{PT} \end{array}\right]}^{t}$ is the vector of additive genetic effect for t traits, and ${\left[\begin{array}{cccc} e_{NP} & e_{NG} & e_{HGW} & e_{PT} \end{array}\right]}^{t}$ is the vector of model residuals for t traits. The ${\left[\begin{array}{cccc} g_{NP} & g_{NG} & g_{HGW} & g_{PT} \end{array}\right]}^{t}$ and ${\left[\begin{array}{cccc} e_{NP} & e_{NG} & e_{HGW} & e_{PT} \end{array}\right]}^{t}$ vectors assumed a multivariate Gaussian distribution of $N(0,\Sigma_{g}⊗G)$ and $N(0,\Sigma_{e}⊗I)$, respectively, where $\Sigma_{g}$ and $\Sigma_{e}$ are the t×t covariance matrices of additive genetic effect and model residuals, respectively, ⊗ indicates the Kronecker product, and G and I are the genomic relationship matrix and the identity matrix, respectively. The G matrix was estimated as **G =** $WW^{\prime }/2\sum_{j=1}^{m}p_{j}(1-p_{j})$, where W is the centered SNP marker matrix [97], $p_{j}$ is the allele frequency of the jth marker, and m is the total number of markers. A non-informative prior was assigned to the vector of ${\left[\begin{array}{cccc} b_{NP} & b_{NG} & b_{HGW} & b_{PT} \end{array}\right]}^{t}$. The $\Sigma_{g}$ and $\Sigma_{e}$ matrices followed an inverse Wishart prior distribution, $W_{t}^{-1}(S,\nu )$, where S and ν are hyperparameters associated with scale and degree of freedom, respectively.

The Markov Chain Monte Carlo (MCMC) approach was used with the Gibbs sampling algorithm to obtain marginal posterior densities. Convergence analysis was performed using the boa R package [98] within the R software environment version 4.5.1 [96]. The MCMC included 3,000,000 iterations, with a burn-in of 300,000 and a thinning rate of 50, yielding 54,000 MCMC samples for inference. Convergence analysis included autocorrelations (Tables S1 and S3) and Geweke tests (Tables S2 and S4).

### 4.4. Bayesian Networks

The Bayesian network (BN) was used to assess the relationships among the traits. A Bayesian network can be viewed as a graphical model based on a directed acyclic graph, where nodes (or vertices) represent random variables and arcs (or edges) represent probabilistic dependencies between them [99,100]. In general, a directed acyclic graph is formed by nodes connected by directed edges, where the probability that an edge exists between nodes (strength) and the probability that the edge has a particular direction (direction) can be estimated.

The hill-climbing (HC) algorithm, implemented in the bnlearn R package [101], was used to estimate a phenotypic network. The HC algorithm finds the best structure of the network by removing arcs and changing their directions. For each edge removal, the Bayesian information criterion (BIC) was computed to infer its relative contribution to the overall BIC of the network. A total of 50,000 bootstrap samples were used to define the strength and direction of each arc. We used the criteria of direction > 50% and strength ≥ 80% to select high-confidence relationships.

### 4.5. Multi-Trait Association Analysis (MTM-GWAS)

MTM-GWAS modeling was performed using the SNP Snappy strategy [102] implemented in the WOMBAT program [103].

(3) $$\left[\begin{array}{c} y_{NP}^{*}\\ y_{NG}^{*}\\ y_{HGW}^{*}\\ y_{PT}^{*} \end{array}\right]=\left[\begin{array}{cccc} {W_{j}}_{NP} & 0 & 0 & 0\\ 0 & {W_{j}}_{NG} & 0 & 0\\ 0 & 0 & {W_{j}}_{HGW} & 0\\ 0 & 0 & 0 & {W_{j}}_{PT} \end{array}\right]\left[\begin{array}{c} {s_{j}}_{NP}\\ {s_{j}}_{NG}\\ {s_{j}}_{HGW}\\ {s_{j}}_{PT} \end{array}\right]+\left[\begin{array}{cccc} Z_{NP} & 0 & 0 & 0\\ 0 & Z_{NG} & 0 & 0\\ 0 & 0 & Z_{HGW} & 0\\ 0 & 0 & 0 & Z_{PT} \end{array}\right]\left[\begin{array}{c} g_{NP}\\ g_{NG}\\ g_{HGW}\\ g_{PT} \end{array}\right]+\left[\begin{array}{c} e_{NP}\\ e_{NG}\\ e_{HGW}\\ e_{PT} \end{array}\right]$$

where ${\left[\begin{array}{cccc} y_{NP}^{*} & y_{NG}^{*} & y_{HGW}^{*} & y_{PT}^{*} \end{array}\right]}^{t}$ is the vector of scaled phenotypes of adjusted t traits, ${W_{j}}_{NP}$, ${W_{j}}_{NG}$, ${W_{j}}_{HGW}$, and ${W_{j}}_{PT}$ are the SNP vector matrices for the jth marker associating ${s_{j}}_{NP}$, ${s_{j}}_{NG}$, ${s_{j}}_{HGW}$, and ${s_{j}}_{PT}$ to $y_{NP}^{*}$, $y_{NG}^{*}$, $y_{HGW}^{*}$, and $y_{PT}^{*}$, and ${\left[\begin{array}{cccc} {s_{j}}_{NP} & {s_{j}}_{NG} & {s_{j}}_{HGW} & {s_{j}}_{PT} \end{array}\right]}^{t}$ is the vector of the jth SNP marker effect for t traits. $Z_{NP}$, $Z_{NG}$, $Z_{HGW}$, and $Z_{PT}$ are the incidence matrices associating $g_{NP}$, $g_{NG}$, $g_{HGW}$, and $g_{PT}$ with $y_{NP}^{*}$, $y_{NG}^{*}$, $y_{HGW}^{*}$, and $y_{PT}^{*}$. The covariance structure was the same as shown earlier. The SNP effects, ${\left[s_{NP},s_{NG},s_{HGW},s_{PT}\right]}^{t}$, were obtained by fitting a single SNP, one at a time, for each trait. A t statistic was used to obtain p-values: $t_{ij}=s_{j}/se(s_{j})$, where s is the point estimate of the jth SNP effect and $se(s_{j})$ is its standard error. The *p*-values were then corrected for multiple testing using the false discovery rate (FDR) procedure, and q-values were considered significant at an FDR threshold of 0.01 [104]. The SNP dosage matrix has also been corrected for the population structure, where 23 principal components were used, resulting in approximately 80% of the genetic variability.

### 4.6. Structural Equations Modeling GWAS (SEM-GWAS)

MTM-GWAS modeling was performed using the SNP Snappy strategy [102] implemented in the WOMBAT program [103].

(4) $$\left[\begin{array}{c} y_{NP}^{*}\\ y_{NG}^{*}\\ y_{HGW}^{*}\\ y_{PT}^{*} \end{array}\right]=\left[\begin{array}{cccc} {W_{j}}_{NP} & 0 & 0 & 0\\ 0 & {W_{j}}_{NG} & 0 & 0\\ 0 & 0 & {W_{j}}_{HGW} & 0\\ 0 & 0 & 0 & {W_{j}}_{PT} \end{array}\right]\left[\begin{array}{c} {s_{j}}_{NP}\\ {s_{j}}_{NG}\\ {s_{j}}_{HGW}\\ {s_{j}}_{PT} \end{array}\right]+\left[\begin{array}{cccc} Z_{NP} & 0 & 0 & 0\\ 0 & Z_{NG} & 0 & 0\\ 0 & 0 & Z_{HGW} & 0\\ 0 & 0 & 0 & Z_{PT} \end{array}\right]\left[\begin{array}{c} g_{NP}\\ g_{NG}\\ g_{HGW}\\ g_{PT} \end{array}\right]+\left[\begin{array}{c} e_{NP}\\ e_{NG}\\ e_{HGW}\\ e_{PT} \end{array}\right]$$

where ${\left[\begin{array}{cccc} y_{NP}^{*} & y_{NG}^{*} & y_{HGW}^{*} & y_{PT}^{*} \end{array}\right]}^{t}$ is the vector of scaled phenotypes of adjusted t traits, ${W_{j}}_{NP}$, ${W_{j}}_{NG}$, ${W_{j}}_{HGW}$, and ${W_{j}}_{PT}$ are the SNP vector matrices for the jth marker associating ${s_{j}}_{NP}$, ${s_{j}}_{NG}$, ${s_{j}}_{HGW}$, and ${s_{j}}_{PT}$ to $y_{NP}^{*}$, $y_{NG}^{*}$, $y_{HGW}^{*}$, and $y_{PT}^{*}$, and ${\left[\begin{array}{cccc} {s_{j}}_{NP} & {s_{j}}_{NG} & {s_{j}}_{HGW} & {s_{j}}_{PT} \end{array}\right]}^{t}$ is the vector of the jth SNP marker effect for t traits. $Z_{NP}$, $Z_{NG}$, $Z_{HGW}$, and $Z_{PT}$ are the incidence matrices associating $g_{NP}$, $g_{NG}$, $g_{HGW}$, and $g_{PT}$ with $y_{NP}^{*}$, $y_{NG}^{*}$, $y_{HGW}^{*}$, and $y_{PT}^{*}$. The covariance structure was the same as shown earlier. The SNP effects, ${\left[s_{NP},s_{NG},s_{HGW},s_{PT}\right]}^{t}$, were obtained by fitting a single SNP, one at a time, for each trait. A t statistic was used to obtain p-values: $t_{ij}=s_{j}/se(s_{j})$, where s is the point estimate of the jth SNP effect and $se(s_{j})$ is its standard error. The *p*-values were then corrected for multiple testing using the false discovery rate (FDR) procedure, and q-values were considered significant at an FDR threshold of 0.01 [104]. The SNP dosage matrix has also been corrected for the population structure, where 23 principal components were used, resulting in approximately 80% of the genetic variability.

### 4.7. Pathway Enrichment Analyses

We selected the relevant SNPs using the *p*-value (<0.01) obtained from MTM-GWAS (no window size was used). We used the *Glycine max* genomic database available at Phytozome [105] (https://phytozome-next.jgi.doe.gov/jbrowse/index.html?data=genomes%2FGmax_Wm82_a2_v1&loc=Chr01%3A5673531..51138829&tracks=Transcripts%2CAlt_Transcripts%2CPASA_assembly%2CBlastx_protein%2CBlatx_Fabidae%2CBlatx_BasalEmbryophyte%2CBlatx_BasalMalvidae&highlight=) (accessed on 13 June 2024) to assess the functionality of each identified gene and the enriched gene ontology (GO) terms.

## 5. Conclusions

This study presents the first application of SEM-GWAS in soybeans to dissect the genetic architecture of complex, interrelated traits. By partitioning SNP effects for NP, NG, HGW, and PT, we identified QTLs with defined biological functions and quantified both direct and indirect genetic influences. Most genetic effects were direct, but HGW showed a moderate indirect contribution through SNPs influencing NG. These results provide new insight into how trait networks shape yield components and highlight the value of SEM-GWAS for uncovering pathways that conventional univariate or multivariate GWAS cannot capture. The approach offers a practical framework for soybean breeding, enabling more precise selection strategies and potentially greater genetic gain.

## Figures and Tables

**Table 1:** Descriptive statistics for soybeans’ morphological and productivity traits.

Trait	Mean	SD
NP ($pods.{plant}^{-1}$)	53.47	11.72
NG ($grains.{plant}^{-1}$)	111.40	22.54
HGW ($g.{100\;grains}^{-1}$)	14.57	3.04
PT (mm)	6.41	0.87

Means and standard deviations (SDs) for number of pods (NP), number of grains (NG), hundred-grain weight (HGW), and pod thickness (PT), measured in 96 *Glycine max* (L.) Merrill genotypes.

**Table 2:** Posterior means of the genomic heritabilities (diagonal), residual (lower triangular), and genomic (upper triangular) correlations of four traits in the soybeans, with posterior standard deviations in parentheses.

	NP	NG	HGW	PT
**NP**	0.89 (0.73, 1.00)	**0.96 (0.82, 1.00)**	**−0.84 (−0.99, −0.55)**	**−0.54 (−0.78, −0.28)**
**NG**	−0.47 (−0.99, 0.84)	0.79 (0.44, 1.00)	**−0.88 (−0.99, −0.68)**	**−0.57 (−0.80, −0.32)**
**HGW**	0.45 (−0.43, 0.98)	−0.24 (−0.78, 0.76)	0.39 (0.14, 0.67)	**0.61 (0.35, 0.83)**
**PT**	0.17 (−0.46, 0.69)	−0.04 (−0.51, 0.57)	**0.59 (0.35, 0.80)**	0.45 (0.24, 0.66)

Residual (lower triangular) and genomic (upper triangular) correlations and heritabilities in the narrow sense (diagonal) and their respective HPD (95% highest probability density) in parenthesis for the number of pods (NP), number of grains (NG), hundred-grain weight (HGW), and pod thickness (PT). Significant correlations are highlighted in bold (HPD without the 0 in the interval).

**Table 3:** Bayesian network score from the Bayesian information criterion (BIC).

BIC (a)	Path	BIC (b)
−907.3641	NP → NG	−35.8808
	NG → HGW	−13.1780
	HGW → PT	−25.9670

(a) The Bayesian information criterion score (BIC) of the general path network; (b) the BIC scores for each path. NP: number of pods; NG: number of grains; HGW: hundred-grain weight; PT: pod thickness.

**Table 4:** Structural coefficient estimates derived from the structural equation models.

Path	Path Coefficient (λ)
NP → NG	0.00006
NG → HGW	−0.05450
HGW → PT	0.00697

Estimates of the structural coefficients (λs) according to the interrelationship structure estimated by the Bayesian network. NP: number of pods; NG: number of grains; HGW: hundred-grain weight; PT: pod thickness.

## Data Availability

The data supporting the findings of this study are available from three of the authors, Nizio Fernando Giasson, Gaspar Malone, and Felipe Lopes da Silva, upon request.

## Supplementary Materials

The following supporting information can be downloaded at: https://zenodo.org/records/16986530 (accessed on 14 June 2025). Figure S1: Gene ontology (GO) terms for the number of pods. SNP obtained from multi-trait association analysis (MTM-GWAS) considering a *p*-value < 0.01.; Figure S2: Gene ontology (GO) terms for the number of grains. SNP obtained from multi-trait association analysis (MTM-GWAS) considering a *p*-value < 0.01.; Table S1: Lags and autocorrelations for genetic chain.; Table S2: Geweke Convergence Diagnostic for genetic chain considering fraction in first window = 0.1 and fraction in last window = 0.5.; Table S3: Lags and autocorrelations for residual chain.; Table S4: Geweke Convergence Diagnostic for residual chain considering fraction in first window = 0.1 and fraction in last window = 0.5.; Table S5: Functional annotation of SNPs (single nucleotide polymorphisms) with significant associations (q < 0.01), chromosome, and position associated for number of reproductive nodes (NRN) and yield (YL).
